# Eosinophilic myocarditis: a diagnostic challenge and treatment dilemma—a case report

**DOI:** 10.1093/ehjcr/ytae418

**Published:** 2024-08-08

**Authors:** Zaid Ammouri, Sami Belkouchia, Ibtissam Rezzouk, Salma Moussaoui, Rachida Habbal

**Affiliations:** Department of Cardiology, Ibn Rochd University Hospital, Casablanca 20503, Morocco; Department of Cardiology, Ibn Rochd University Hospital, Casablanca 20503, Morocco; Department of Cardiology, Ibn Rochd University Hospital, Casablanca 20503, Morocco; Department of Central Radiology, Ibn Rochd University Hospital, Casablanca, Morocco; Department of Cardiology, Ibn Rochd University Hospital, Casablanca 20503, Morocco

**Keywords:** Myocarditis, Hypereosinophilic syndrome, Minoca, Case report, Acute coronary syndrome

## Abstract

**Background:**

Eosinophilic myocarditis, a rare and potentially life-threatening condition, can resemble acute coronary syndrome (ACS) and presents diagnostic difficulties.

**Case report:**

We describe the case of a 32-year-old man initially admitted with ACS-like symptoms, but ultimately diagnosed with eosinophilic myocarditis. The patient presented with intense retrosternal chest pain, significant eosinophilia, and elevated cardiac enzymes. Despite clinical indications suggesting myocardial involvement, an endomyocardial biopsy was not performed due to the patient’s reluctance. Non-invasive imaging and clinical findings led to the presumptive diagnosis of eosinophilic myocarditis. The patient was treated with high-dose corticosteroids and immunosuppressive therapy, resulting in clinical improvement.

**Discussion:**

Our report highlights the importance of considering eosinophilic myocarditis and hypereosinophilic syndrome when evaluating patients with chest pain and hypereosinophilia. It emphasizes the subtleties of diagnosis and the critical need for early identification and appropriate treatment to improve prognosis in cases of eosinophilic myocarditis. This case underscores the diverse clinical manifestations of myocarditis and the essential need for a comprehensive diagnostic approach in the presence of chest pain and hypereosinophilia.

**Learning points**

- Eosinophilic myocarditis, a rare and potentially life-threatening condition, can resemble acute coronary syndrome and presents diagnostic difficulties.
- Cardiac involvement occurs in around 20% of cases of hypereosinophilic syndrome and marks a critical phase.

## Introduction

Eosinophilic myocarditis (EM) is uncommon, accounting for approximately 0.5–5% of all cases of myocarditis.

Eosinophilic myocarditis represents a rare yet clinically significant condition characterized by myocardial inflammation and infiltration of eosinophils, leading to cardiac dysfunction and potentially life-threatening complications.^1^

This condition, first identified by Wilhelm Löffler in 1936,^2^ remains under-represented in myocardial disease literature, with its pathophysiology rooted in both allergic and non-allergic mechanisms ranging from drug hypersensitivity to autoimmune disorders.^1–3^ Eosinophilic myocarditis’ histological hallmark is myocardial eosinophil presence, which can progress through stages of necrosis, thrombosis, and fibrosis, each linked to specific disease durations and complications such as heart failure, arrhythmias, and even sudden death.^2,3^

We report the case of a 32-year-old man who was initially admitted with symptoms suggestive of acute coronary syndrome (ACS). His presentation underscores the importance of considering EM in differential diagnoses, especially when there is evidence of eosinophilia.

Eosinophilic myocarditis is often associated with hypereosinophilic syndrome (HES), a condition characterized by chronic hypereosinophilia leading to multi-organ tissue damage. Cardiac involvement in HES, although occurring in approximately 20% of cases, marks a critical and potentially life-threatening phase of the disease.

This case epitomizes the complex intersection of EM and HES, emphasizing the need for awareness and understanding of these conditions’ pathophysiological and clinical nuances. Our report delves into the diagnostic complexities and highlights the significance of a comprehensive diagnostic approach in patients presenting with chest pain and hypereosinophilia.

## Summary figure

**Figure:**
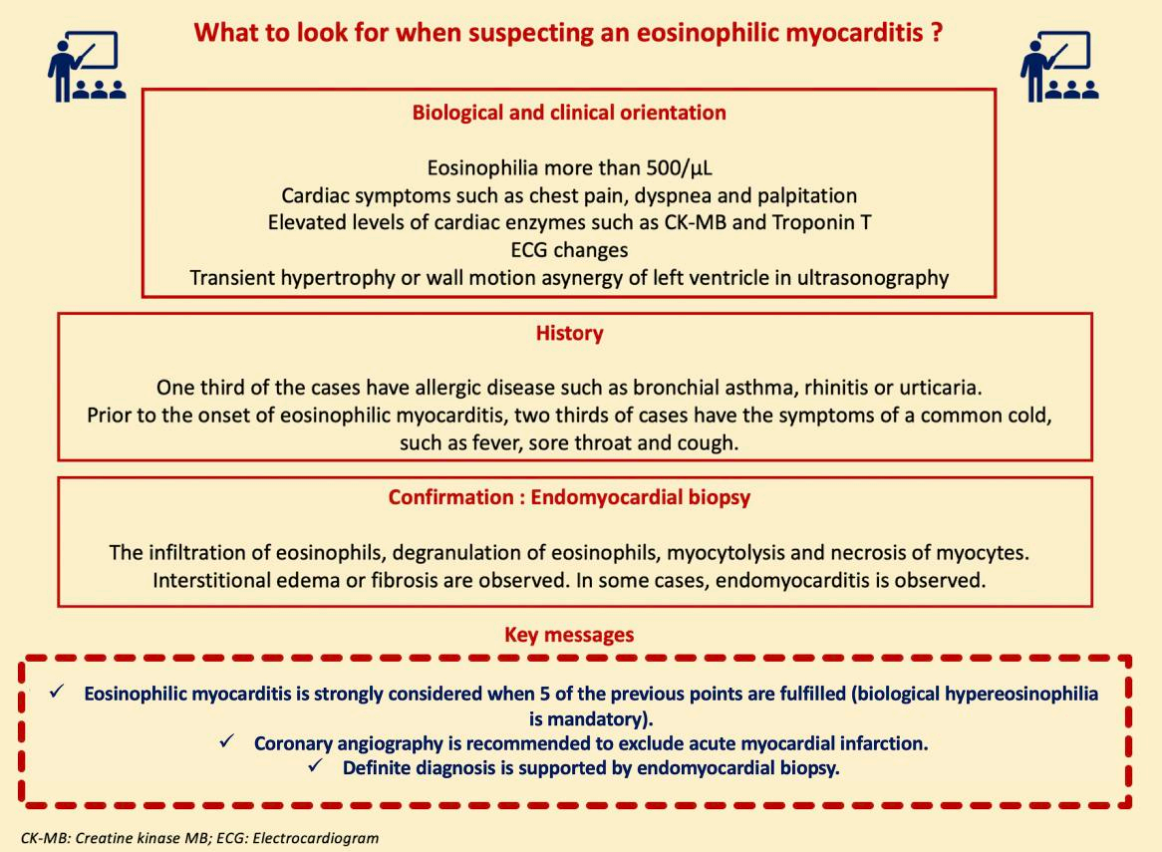


## Case report

The patient was a 32-year-old man with a history of active smoking, which he had managed to stop 1 month prior to admission.

It is important to note that the patient had been followed for several months for respiratory symptoms. These symptoms included episodes of wheezing dyspnoea and cough, treated with bronchodilators and inhaled corticosteroids. In addition, the patient had noted the emergence of non-pruritic, erythematous maculopapular lesions scattered across his trunk and limbs, which had not been present previously. The patient had also experienced significant weight loss over the preceding months.

The patient presented to hospital with a chief complaint of intense retrosternal chest pain without radiation, characterized by its sudden onset and associated symptoms of diaphoresis and breathlessness. Importantly, the patient had no history of cardiovascular disease or other significant medical problems.

### Clinico-biological presentation

On initial assessment, the patient presented with low blood pressure (100/50 mmHg), heart rate of 110 b.p.m., respiratory rate of 20 b.p.m., and oxygen saturation of 98% on room air.

Cardiac auscultation was normal, with no murmurs or rubs. The lungs showed clear breath sounds bilaterally. The electrocardiogram (ECG) displayed diffuse ST depression, suggesting widespread myocardial ischaemia or injury.

Blood test results showed a significant elevation of troponin I to 890 ng/mL (reference < 10 ng/mL), a blood count with a high eosinophilia of 7500 cells/μL among 18 000 white blood cells (reference range 4000–11 000 cells/μL), and normal values for renal and liver function. At presentation, the C-reactive protein (CRP) level was significantly elevated at 28 mg/L (reference < 5 mg/L), indicating the presence of an acute inflammatory response.

### Additional examinations

**Figure 1:**
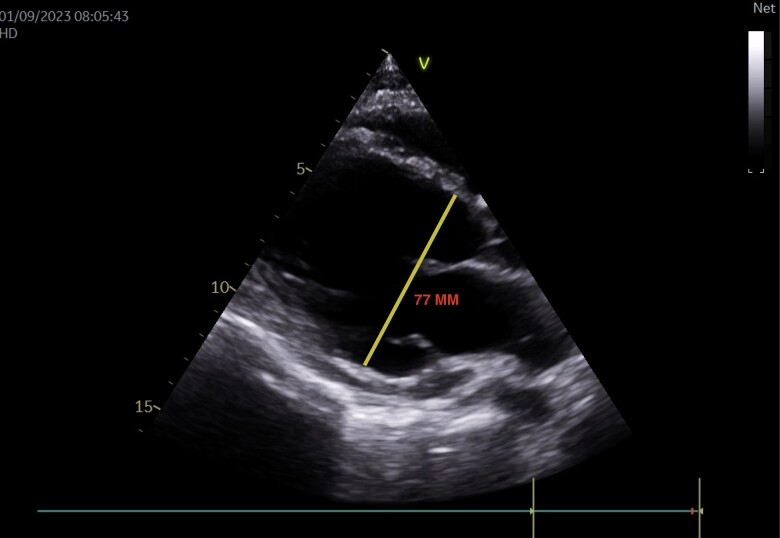
Parasternal long-axis echocardiographic section showing a dilated left ventricle with a telediastolic diameter of 77 mm.

**Figure 2:**
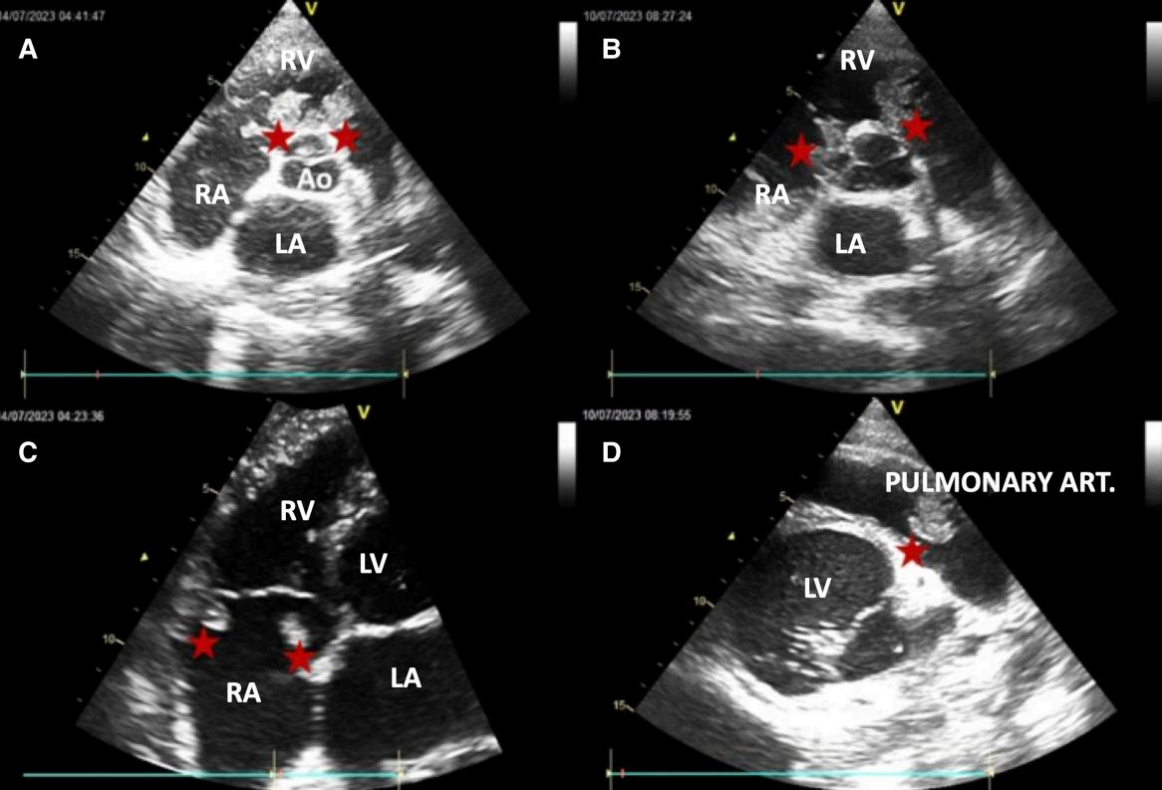
Echocardiographic sections showing thrombi. (*A* and *B*) Short-axis parasternal section passing through the root of the great vessels. (*C*) Apical four cavities centred on the RV. (*D*) Modified short-axis parasternal section centred on the trunk of the pulmonary artery. Red stars, thrombi; RA, right atrium; LA, left atrium; RV, right ventricle; LV, left ventricle; Ao, aorta.

Echocardiography revealed a dilated and hypokinetic heart with significant dilatation of the left ventricle, leading to severe impairment of cardiac function [left ventricular ejection fraction (LVEF) estimated at 27%]. Multiple thrombi were detected in the heart chambers, notably attached to the valves, in particular the pulmonary valve and both atria (*Figures 1* and *2*).

Initial coronary angiography ruled out significant coronary artery disease, showing no evidence of coronary stenosis or occlusion.

**Figure 3:**
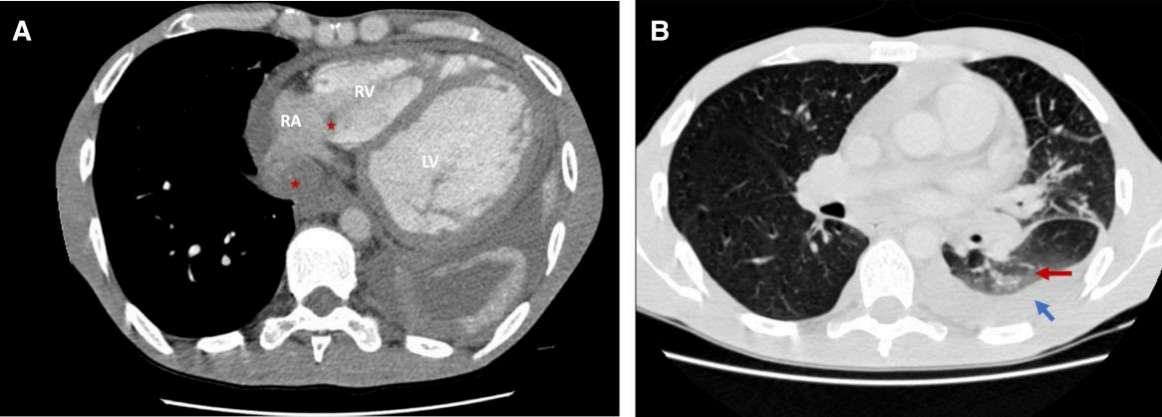
(*A*) Axial thoracic scan showing the same thrombi seen on transthoracic echocardiography (red stars) as well as cardiomegaly and dilatation of the left ventricle. (*B*) Axial thoracic scan showing ground glass (red arrow) opacities and pleural effusion (blue arrow). RA, right atrium; RV, right ventricle; LV, left ventricle.

A thoracic–abdominal–pelvic computed tomography (CT) scan showed cardiomegaly and intracardiac thrombi. In the lungs, the CT scan revealed signs of eosinophilic lung involvement, evidenced by ground glass opacities and bilateral consolidations (*Figure 3A* and *B*).

**Figure 4:**
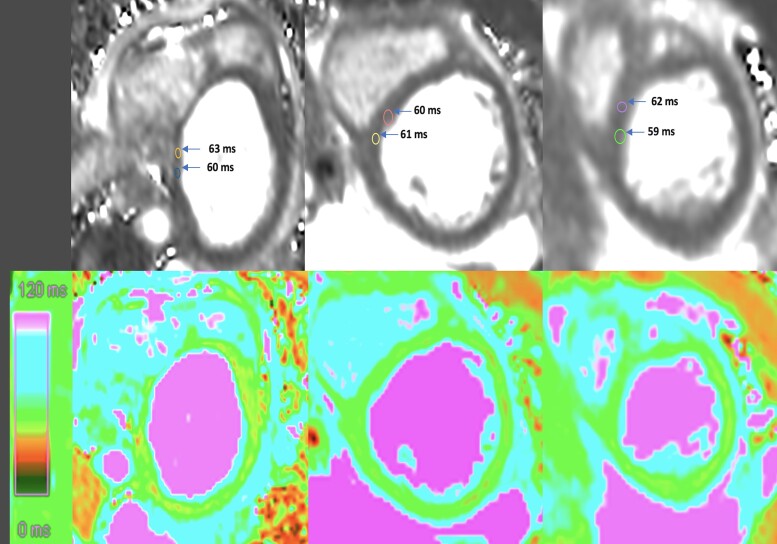
T2 mapping sequences: black and white and colour images (basal, mid, and apical sections) revealed elevated zones of T2 mapping particularly in septal wall indicative of diffuse myocardial oedema.

**Figure 5:**
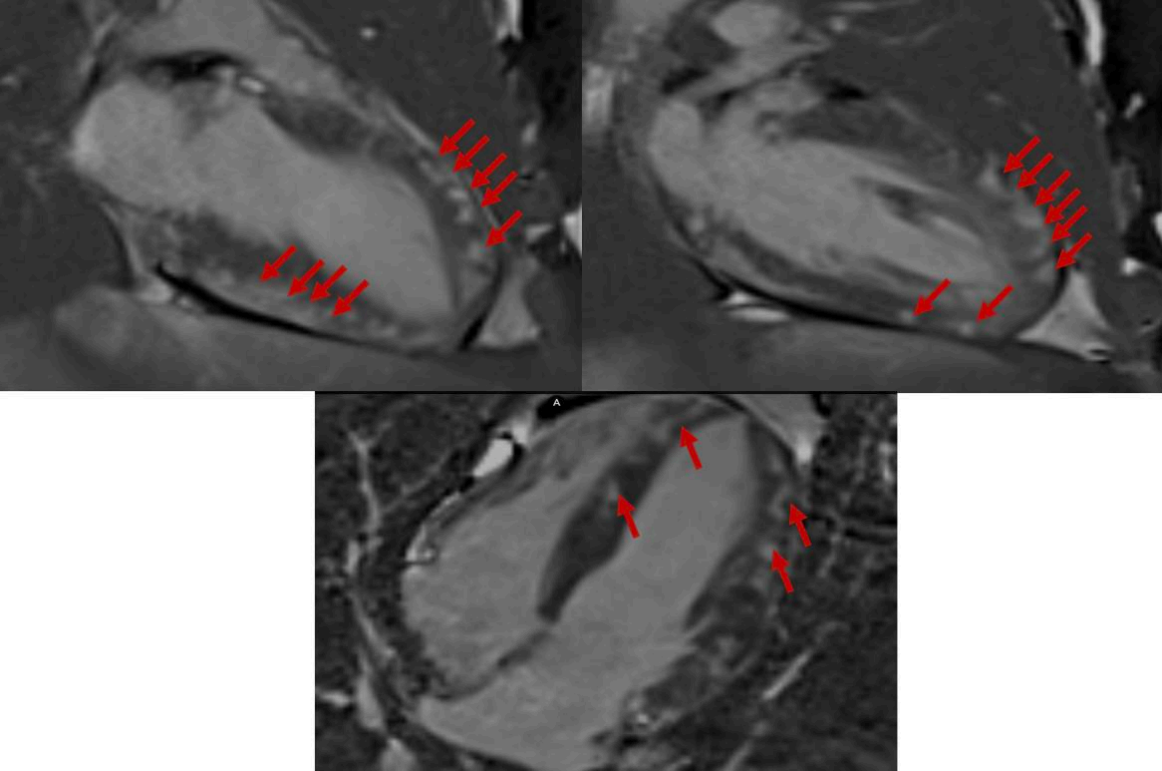
Late gadolinium enhancement cardiac magnetic resonance two-chamber and four-chamber sequences showing non-segmented subepicardial patchy enhancement patterns in favour of myocarditis. The late gadolinium enhancement is more marked in the anterior, anteroseptal, and inferior walls. Red arrows: late gadolinium enhancement.

Cardiac magnetic resonance (CMR) was performed using the Lake Louise criteria (LLC) for myocarditis diagnosis. Cardiac magnetic resonance demonstrated myocardial oedema with increased T2 signal intensity (mean of 60 ms in the septal wall; normal range 45–50 ms for a 1.5 T magnetic resonance imaging machine). Late gadolinium enhancement (LGE) sequences in both two-chamber and four-chamber views showed non-segmental, subepicardial patchy enhancement, particularly in the anterior, anteroseptal, and inferior walls. This pattern of LGE supports the diagnosis of myocarditis (*Figures 4* and *5*).

### Immunological and tumour screening

Extensive immunological and tumour investigations were performed to explore the aetiology of this patient’s HES. However, no evidence was found to identify the origin or underlying cause of the syndrome, further complicating the diagnostic conundrum. Serological tests for infectious agents, including viral, bacterial, and parasitic pathogens, were negative. Autoimmune serologies, such as antinuclear antibodies and rheumatoid factor, were within normal limits. Evaluation for vasculitis, including inflammatory markers such as erythrocyte sedimentation rate and CRP, showed no evidence of systemic inflammation or vasculitic processes. Bone marrow assessment revealed normocellular marrow with no evidence of myeloproliferative disorders or haematological malignancies contributing to eosinophilia.

### Skin biopsy

A skin biopsy revealed non-specific dermatitis with mild perivascular lymphocytic infiltration and eosinophils in the dermis. Although the biopsy did not provide definitive diagnostic insights, it helped rule out specific cutaneous manifestations suggestive of systemic vasculitis or other skin disorders, supporting the clinical suspicion of EM.

### Endomyocardial biopsy

An endomyocardial biopsy was considered to confirm the diagnosis of EM. However, the patient declined the procedure due to concerns about its invasiveness. Respecting the patient’s autonomy and given their stable clinical condition, a decision was made to forgo the biopsy. Consequently, the diagnosis of EM remains presumptive, based on clinical presentation and supportive non-invasive imaging findings.

### Therapeutic approach

Upon establishing a strong presumptive diagnosis of HES, we initiated a comprehensive treatment regimen. Prednisone was dosed at 1 mg/kg/day (starting at 60 mg daily), tapering over 6 weeks to a maintenance dose. For thrombosis management, the patient was transitioned from low molecular weight heparin to acenocoumarol, with dosing adjusted to achieve a therapeutic international normalised ratio (INR) of 2–3. The heart failure regimen included bisoprolol (2.5 mg daily), perindopril (4 mg daily), and dapagliflozin (10 mg daily). Furosemide (Lasilix) at 40 mg daily was used to manage fluid overload.

### Evolution

Our therapeutic approach yielded significant improvements in cardiorespiratory symptoms and skin involvement. The patient’s dyspnoea and chest pain markedly decreased, and the erythematous maculopapular lesions on his trunk and limbs resolved. Echocardiographic monitoring showed an increase in LVEF from 27% at baseline to 38% after 6 months. Despite the overall clinical improvement, the INR was challenging to maintain within the therapeutic range, leading to the persistence of thrombi, particularly in the pulmonary sub-valvular apparatus. Consequently, anti-vitamin K–based anticoagulant therapy was continued. A follow-up consultation is scheduled for 2 months to reassess LVEF and thrombi evolution, emphasizing the importance of ongoing multidisciplinary care.

## Discussion

This case report highlights a 32-year-old man with EM initially presenting with ACS-like symptoms. The key learning point from this case is the importance of considering EM in patients with chest pain and hypereosinophilia, as early diagnosis and treatment are crucial for improving prognosis.

Eosinophilic myocarditis represents a rare yet clinically significant condition characterized by myocardial inflammation and infiltration of eosinophils, leading to cardiac dysfunction and potentially life-threatening complications.^1^ First identified by Wilhelm Löffler in 1936,^2^ EM remains under-represented in myocardial disease literature, with its pathophysiology rooted in both allergic and non-allergic mechanisms ranging from drug hypersensitivity to autoimmune disorders.^1–3^ Eosinophilic myocarditis’ histological hallmark is the presence of myocardial eosinophils, which can progress through stages of necrosis, thrombosis, and fibrosis, each linked to specific disease durations and complications such as heart failure, arrhythmias, and sudden death.^2,3^

The acute stage of EM involves the formation of mural thrombi, frequently affecting both ventricles, ventricular outflow tracts, and sub-valvular regions, with the risk of atrioventricular valve regurgitation.^4^ Manifestations of HES related to cardiac involvement frequently include heart failure and intracardiac thrombi, with myocardial ischaemia, arrhythmias, and occasionally pericarditis also noted.^5^

In a review of 65 HES cases, dyspnoea was the most prevalent symptom, occurring in 60% of patients. Furthermore, congestive heart failure was evident in 75% of these patients, and a minority exhibited pericarditis.^5^

Though rare, myocardial infarction associated with EM can occur due to embolic events from endomyocardial fibrosis and thrombosis within the left ventricular outflow tract.^6^ This case underscores the diagnostic complexities of EM, especially its presentation, which can closely resemble that of ACS, underscoring the necessity for careful differential diagnosis.

The principal imaging techniques utilized for cardiac assessment in EM include echocardiography, CMR, and endomyocardial biopsy. Common ECG anomalies in EM are typically non-specific, often presenting as T-wave inversions, atrial enlargement, ventricular hypertrophy, incomplete right bundle branch block, and left axis deviation.^3^ Further ECG irregularities may include premature ventricular contractions, attenuated R-wave progression, and non-specific ST-T changes, along with first-degree atrioventricular block.^5^

Cardiac magnetic resonance, in particular, has gained prominence as an invaluable non-invasive tool in diagnosing EM, excelling in sensitivity and specificity for identifying ventricular thrombi when compared with echocardiographic methods.^7^ Moreover, LGE in CMR is instrumental in detecting myocardial fibrosis and inflammation.^8^ The LLC have become pivotal in the CMR evaluation of EM, providing a standardized set of parameters for identifying myocardial tissue changes indicative of inflammation and injury, enhancing the accuracy of diagnosis.

These imaging modalities are not just diagnostic tools but are integral to the management and prognostication of EM. They enable clinicians to non-invasively assess the extent of cardiac involvement in HES, guiding therapeutic decision-making and patient care.

The approach to managing HES is multifaceted, with corticosteroids forming the mainstay of treatment, particularly for the lymphocytic variant (L-HES). For those with the myeloproliferative variant harbouring the FIP1L1-PDGFRA (FP) fusion gene, which instigates abnormal tyrosine kinase activity, imatinib, a tyrosine kinase inhibitor, has been notably effective, with approximately 88% of FP-positive patients responding favourably.^1^ Corticosteroids play a crucial role by reducing myocardial inflammation and curbing eosinophilic activity, addressing the core inflammatory process at the heart of EM.

In instances where corticosteroids are insufficient or the condition is resistant, additional immunosuppressants such as azathioprine, cyclophosphamide, or mycophenolate mofetil are considered to bolster the immune suppression and curb eosinophil proliferation.^9^ The selection of these therapies is personalized, taking into account patient-specific factors like drug tolerance and potential side effects.

Standard heart failure treatments—diuretics, beta-blockers, angiotensin-converting enzyme inhibitors, and sodium–glucose cotransporter 2 inhibitors—are integrated to manage symptoms and improve cardiac function, guided by heart failure management guidelines which emphasize their synergistic benefits in enhancing cardiovascular health.^10^

Moreover, anticoagulation therapy is critical for thrombus management, a frequent complication in EM, to diminish the risk of thromboembolic incidents. The preference for vitamin K antagonists in this context is based on their proven effectiveness in thrombus prevention, particularly in patients with intracardiac thrombi.^11^ The overarching goal of the treatment regimen is to achieve a balance between efficacy and safety, tailored to the individual’s health profile and tolerance.

Ultimately, the therapeutic strategy is comprehensive, aiming to suppress the inflammatory activity, manage cardiac dysfunction, and prevent complications, thereby striving to improve the prognosis for patients with EM.

## Conclusion

In conclusion, our case highlights the complexity of EM, its ability to mimic ACS, and the difficulties in diagnosing an underlying HES. The presence of thrombi in the heart chambers adds a further layer of complexity to the clinical picture. This case aligns with the findings in the literature, highlighting the importance of early recognition and a comprehensive diagnostic approach to EM in young adults.

## Lead author biography


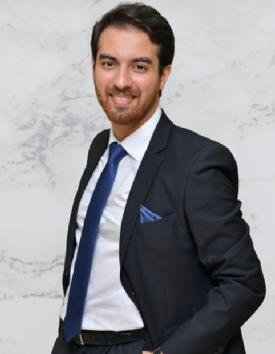


Dedicated to the medical field, I am on the path to becoming a cardiologist, infusing boundless motivation into patient care and my passion for cardiology. Dynamic and proactive, I excel in crisis management and thrive in collaborative environments. Beyond medicine, life’s richness unfolds in my diverse interests—sports fuel my excitement, cinema enchants me, travel broadens my horizons, literature captivates, cuisine is an art, and music sets the rhythm of my days. Engaged in associative work, I find purpose in making a positive impact within my community. In all pursuits, be it medical or personal, my dynamic approach, creativity, and commitment shine, painting a vibrant tapestry that defines my journey.

## Data Availability

As this manuscript is a case report, there are no data sets or data repositories associated with it. All relevant data pertaining to the case are included within the manuscript. This statement clarifies that as a case report, there are no additional data sets or data repositories associated with the study, and all pertinent data are contained within the manuscript itself.

## Notes

**Consent:** The patient involved in this case report has provided consent for the publication of their case details, including any associated images and clinical information. He understands that all personal identifiers will be removed to maintain anonymity and confidentiality. The patient acknowledges that his participation in this publication is voluntary and that he has been given the opportunity to review and approve the content. The patient understands that this case report will be made publicly available and may be accessed by individuals worldwide. Therefore, he grants permission for the publication of this case report in compliance with the guidelines set forth by the Committee on Publication Ethics (COPE). This statement confirms that the patient has provided informed consent for the publication of their case report and complies with the COPE guidelines.

**Funding:** None declared.
